# Sleep-dependent prospective memory consolidation is impaired with aging

**DOI:** 10.1093/sleep/zsab069

**Published:** 2021-03-23

**Authors:** Ruth L F Leong, June C Lo, Michael W L Chee

**Affiliations:** Centre for Sleep and Cognition, Human Potential Program, Yong Loo Lin School of Medicine, National University of Singapore, Singapore

**Keywords:** memory, consolidation, slow-wave sleep, aging

## Abstract

**Study Objectives:**

Existing literature suggests that sleep-dependent memory consolidation is impaired in older adults but may be preserved for personally relevant information. Prospective memory (PM) involves remembering to execute future intentions in a timely manner and has behavioral importance. As previous work suggests that N3 sleep is important for PM in young adults, we investigated if the role of N3 sleep in PM consolidation would be maintained in older adults.

**Methods:**

Forty-nine young adults (mean age ± SD: 21.8 ± 1.61 years) and 49 healthy older adults (mean age ± SD: 65.7 ± 6.30 years) were randomized into sleep and wake groups. After a semantic categorization task, participants encoded intentions comprising four related and four unrelated cue-action pairs. They were instructed to remember to perform these actions in response to cue words presented during a second semantic categorization task 12 h later that encompassed either daytime wake (09:00 am–21:00 pm) or overnight sleep with polysomnography (21:00 pm–09:00 am).

**Results:**

The significant condition × age group × relatedness interaction suggested that the sleep benefit on PM intentions varied according to age group and relatedness (*p* = 0.01). For related intentions, sleep relative to wake benefitted young adults’ performance (*p* < 0.001) but not older adults (*p* = 0.30). For unrelated intentions, sleep did not improve PM for either age group. While post-encoding N3 was significantly associated with related intentions’ execution in young adults (*r* = 0.43, *p* = 0.02), this relationship was not found for older adults (*r* = −0.07, *p* = 0.763).

**Conclusions:**

The age-related impairment of sleep-dependent memory consolidation extends to PM. Our findings add to an existing body of work suggesting that the link between sleep and memory is functionally weakened in older adulthood.

**Statement of Significance**

Prospective memory (PM) is ubiquitous in everyday life and has clinical importance, for example, remembering to take medication on time. In order to clarify whether the benefit of sleep for memory consolidation would be preserved in older adults if study material has future relevance, we compared performance on a PM task across nocturnal sleep and daytime wake intervals for young and older adults. Unlike in young adults, sleep did not improve older adults’ performance on the PM task and there was no relationship between related intentions executed and the amount of N3 obtained. Our findings are in line with the literature reporting age-related changes in sleep’s role in memory and suggest an impairment even when to-be-remembered material incorporates behavioral relevance.

## Introduction

While sleep unequivocally benefits the consolidation of declarative [1–3] and procedural memory [4–6] in young adults, an increasing number of studies have begun to show that in older adults, this sleep effect is significantly reduced or absent [7–9]. However, this may vary according to the nature of the memoranda. For example, sleep-dependent memory consolidation for procedural memory [10–12] appear to be relatively preserved compared to declarative memory which shows the greatest age impairment [7]. Interestingly, declarative memory consolidation may be preserved if older adults perceive study material to be engaging and personally relevant [13]. For example, while no sleep improvements were found for word pairs [14–16], one study showed that sleep versus wake benefited memory for short stories and personal events (e.g. the first conversation one had that morning) in older adults, albeit to a smaller extent than young adults [17].

This may occur because relevance renders memories more salient, improving the synaptic signal to noise ratio such that these representations are preferentially reactivated and strengthened during sleep [18–20]. For example, in young adults, sleep-dependent gains have been found to be greater for material with future relevance, such as words [21] and skills [22] for which participants anticipated a reward, as well as for items for which they expected a future test [23]. With aging, as the brain undergoes atrophy [14] and memory declines, the benefit of sleep on memory may become more dependent on the relevance and salience of the memoranda. However, few studies have used material involving future relevance to contrast sleep effects in younger and older participants.

Here, we investigated age-related differences in the consolidation of prospective memory (PM), a type of memory that involves encoding actions that need to be retrieved upon the later appearance of a specific cue, for example, passing a message to a colleague the moment they arrive at work [24, 25]. To test this in the laboratory, a prospective memory task whereby participants are required to execute an action in response to a cue is typically embedded within an ongoing task (e.g. a semantic categorization task). Successful prospective remembering involves the retrieval of the content of the intention (retrospective component) in a timely manner in response to the correct event (prospective component).

Prospective memory failures account for 50%–80% of all everyday memory complaints [26], highlighting its functional importance. Recent evidence also suggests that memories with richer contextual cues (e.g. “what,” “where,” “when” details) may also be protected against age-related declines in sleep consolidation [27]. Given that the timely execution of intentions necessarily depends on the successful use of environmental and temporal information, we sought to examine whether prospective memory consolidation would be preserved with aging.

In our previous study on young adults [28], we found that compared to daytime wakefulness, a period of overnight sleep benefitted spontaneous retrieval of intentions comprising related cue-action pairs (e.g. switching on an alarm in response to the target word “clock”) [29]. In contrast, sleep did not improve the execution of semantically unrelated cue-action pairs (e.g. closing a book in response to “mirror”), suggesting that that sleep may preferentially facilitate intentions comprising pre-existing associations. Moreover, higher execution of related intentions after sleep occurred without additional costs to the ongoing task, indicating a sleep-specific effect on spontaneous retrieval as opposed to the attentionally demanding process of cue monitoring. Duration of post-encoding N3 sleep was positively correlated to the execution of related intentions. Given that slow-wave duration [30] and amplitude of slow oscillations [31, 32] are substantially reduced with aging, it is an open question whether degraded sleep in older adults would affect prospective memory.

Previously, a meta-analysis reported that the effect of sleep on prospective memory in older adults was small and not statistically significant [33]. However, this effect size was based solely on observational studies, as at the time no experiment had been conducted to compare the sleep and wake effects on older adults’ prospective memory. Recently, using a virtual environment paradigm, Rehel et al.’ reported a sleep benefit on older adults’ prospective memory, particularly for related intentions [34]. However, the role of sleep architecture in intention consolidation was not evaluated. To address this gap, we contrasted prospective memory performance after polysomnography-monitored sleep versus wake in both younger and older adults and examined if age differences could be attributed to alterations in the sleep–memory relationship. We hypothesized that the effect of sleep on prospective memory consolidation would be diminished in older adults compared to young adults, and that this would be accompanied by changes in the association with sleep.

## Methods

### Participants

Sixty healthy older adults (age range: 57–77 years) were recruited and compared with 49 young adults (age range: 19–25 years) from a precedent study [28]. Older adult participants were native English speakers recruited from the Singapore-Longitudinal Aging Brain Study [35] or by word of mouth. Participants had no history of significant vascular events (i.e. myocardial infarction, stroke, or peripheral vascular disease), malignant neoplasia of any form, cardiac, lung, liver, or kidney failure, active or inadequately treated thyroid disease and no active neurological or psychiatric conditions. In addition, all participants scored >26 on the Mini-Mental State Examination [36], and >5 on the 15-item modified Geriatric Depression Screening Scale indicating absence of depression symptoms [37]. Participants were screened for symptoms of sleep apnea using the Berlin Questionnaire [38]. All participants provided informed consent, in compliance with a protocol approved by the National University of Singapore Institutional Review Board.

Older adults were randomized equally into sleep and wake groups. In total, 11 participants misunderstood the task instructions as well as indicated that their poor sleep in the laboratory was not representative of their usual sleep. After excluding these 11 participants from all analyses, the final sample consisted of 49 older adults (mean age ± SD: 65.7 ± 6.3 years, 23 males, Table 1) and 49 young adults (mean age ± SD: 21.8 ± 1.61 years, 18 males). Within each age group, sleep and wake groups did not differ in age, gender distribution, consumption of caffeinated beverages per day, level of daytime sleepiness measured by the Epworth Sleepiness Scale [39], or self-reported sleep habits and subjective sleep quality as measured by the Pittsburgh Sleep Quality Index [40] (*p* > 0.26). In line with trends reported elsewhere [41–43], sleep habits differed between age groups. Compared to young adults, older reported sleeping and waking earlier on both weekdays and weekends (*p*s < 0.001). This sleep timing difference was particularly prominent on weekends, resulting in significantly shorter sleep time in older adults on weekends (*p* < 0.001).

**Table 1.** Characteristics of the younger and older adult sleep and wake groups

	Young adults				Older adults				
	Sleep		Wake		Sleep		Wake		
	Mean	SD	Mean	SD	Mean	SD	Mean	SD	*p*
*n*	25	–	24	–	23	–	26	–	–
Gender (no. of males)	6	–	12	–	13	–	10	–	0.27
Age (years)	22.38	1.79	21.76	1.61	65.81	6.14	65.65	6.30	<0.001
Daily caffeine intake (cups)	0.96	0.92	0.85	0.68	0.99	0.78	0.89	0.97	0.94
Epworth Sleepiness Scale	5.48	1.92	4.71	2.40	6.01	2.67	5.89	3.09	0.29
Pittsburgh Sleep Quality Index									
Weekday bedtime^†^	00:02	0.88	00:17	0.88	23:21	0.68	23:11	1.07	<0.001
Weekday wake time^†^	07:52	0.81	08:06	0.78	07:37	0.96	07:30	0.91	<0.001
Weekday TST (h)	7.23	0.73	7.26	0.84	6.67	0.94	6.95	0.86	0.12
Weekend bedtime^†^	00:20	0.99	00:21	0.97	23:18	0.86	23:16	1.00	<0.001
Weekend wake time^†^	08:29	1.28	08:31	0.85	06:40	0.99	06:53	0.93	<0.001
Weekend TST (h)	7.57	0.65	7.61	0.62	6.68	0.52	6.96	0.88	<0.001
Global score	2.46	1.64	2.56	1.56	3.55	1.99	3.50	1.41	0.06

^†^ Values for means in hh:mm.

*p* values from the ANOVA and Chi-squared tests contrasting the four groups are reported. Note. Within each age group, there were no significant contrasts between sleep and wake groups.

### Study protocol

Participants were instructed to adhere to a consistent sleep schedule in the three days prior to the study, and compliance with this instruction was verified with wrist actigraphy (Actiwatch 2, Philips Respironics, Murrysville, PA). The intention encoding and retrieval sessions took place 12 h apart and included either a period of polysomnography-monitored overnight sleep at the laboratory or a period of daytime wakefulness (no napping allowed).

Participants assigned to the sleep group were scheduled to arrive at the laboratory at 21:00 pm. The intention encoding session was conducted at 21:30 pm and lasted for approximately 30–40 min. Polysomnography was applied and participants slept and woke at their habitual sleep timings (within 23:00 pm and 08:00 am). The following morning, the intention retrieval session was performed at 09:30 am.

In the wake group, participants arrived at the laboratory in the morning for the intention encoding session which started at 09:30 am. Upon completion, they were discharged and instructed to engage in their daily routine, but to avoid napping and the consumption of caffeinated food and drinks. Participants were scheduled to return to the laboratory in the evening for the intention retrieval session which took place at 21:30 pm.

Prior to each session, participants were asked to report their level of subjective alertness on the Karolinska Sleepiness Scale (1—very alert, 9—very sleepy, great effort to keep awake). At the end of the final intention retrieval session, participants were debriefed, and a short interview was conducted to probe the strategies they may have used as well as to verify that they had fully understood the requirements of the task. For example, some participants failed to execute their intentions not due to forgetting but because they became confused about the task instructions.

### Prospective memory task

The prospective memory task required participants to remember to perform actions once cue words are encountered while performing an ongoing semantic categorization task [28]. The likelihood of spontaneous retrieval of the action was manipulated by varying the relatedness of the cue word and associated action. Compared to semantically unrelated cue-action pairs (“mirror-close the book”), semantically related cue-action pairs (“phone-unplug earphones”) are more likely to trigger a spontaneous associative retrieval process, bringing the intention reflexively to mind once the cue word is encountered during the semantic categorization task [29].

The intention encoding session involved familiarizing participants with the semantic categorization task. After participants performed this task once, the experimenter explained that in the session 12 h later, they would be asked to perform the semantic categorization task again but would now also have to remember to perform specific actions when specific words are encountered during the task. This constituted the prospective memory task - participants would have to remember to execute these actions in response to the right cue without being reminded by the experimenter.

On each trial of the semantic categorization task, participants had to determine if a word presented on the left of the computer screen was a member of the category word presented to the right of it (“hockey SPORT”). To indicate “yes” or “no” answers, participants pressed “1” and “2” on the keyboard respectively. The semantic categorization task consisted of 144 trials. Performance was indicated by the proportion of trials correctly responded to. Median reaction times (RT) for these correct trials for each participant was derived.

In the intention encoding session, after performing their first semantic categorization task, participants were shown the following instructions: “In addition to this semantic categorization task, we have a secondary interest in your ability to remember to perform future actions. When you return to the lab later, you will perform this task again. This is a list of words, with actions associated with each word. When you see any of these words as you are doing the task, you will need to remember to perform the associated action. When you notice the word, first press ‘Q’ to pause the experiment, then perform the action. If you remember after the trial has already passed, you can still press ‘Q’ and perform the action. After performing the action, carry on with the next trial of the categorization task. Note that the target words will occur as part of the categorization task. They may be present as the word in lowercase letters, or the word in capital letters. You will NOT be reminded of this instruction at the next session.” Participants then proceeded to learn the eight intentions, four of which comprised related cue-action pairs (e.g. switching on an alarm in response to the target word “clock”), and the other four comprising semantically unrelated cue-action pairs (e.g. closing a book in response to “mirror”; see Leong et al. 2019 for full details). Notably, the intentions all had to be physically performed on items placed in the testing room. Participants were given 10 min to study the eight pairs and were required to verbally recall the pairs to the experimenter until all were recalled correctly. All participants achieved 100% accuracy on their first recall attempt.

In the intention retrieval session 12 h later, participants were presented with the semantic categorization task. The experimenter did not remind participants of the prospective memory task. Each cue occurred once within the semantic categorization task and in the same order for all participants. Accuracy on the prospective memory task was quantified by the percentage of cue words correctly responded to within five trials of the semantic categorization task. At the end of the semantic categorization, participants were given a recognition test for the cue words and actions. This allowed us to discern whether prospective memory failures were due to errors in the retrospective component of prospective memory, that is failure to execute the intention due to the cue-action pairing being forgotten, or errors in the prospective component, i.e. missing the opportunity to execute the intention.

### Polysomnography

Electroencephalographic (EEG) signals during overnight sleep were recorded using a six-channel EEG montage (F3-A2, F4-A1, C3-A2, C4-A1, O1-A2 and O2-A1) according to the 10–20 system. Eye movement and muscle tone were recorded through left and right electrooculographic (EOG) and submental electromyographic (EMG) electrodes that are respectively referenced to A2 and A1. The ground and common reference electrodes were placed at Cz and FPz, respectively.

EEG, EOG, and EMG signals were recorded using a Comet Portable EEG system from Grass Technologies (Astro-Med, Inc., West Warwick, RI). The sampling rate and the storage rate were 800 and 200 Hz, respectively. The low-pass and high-pass filters were set at 35 and 0.3 Hz for the EEG signals and 70 and 10 Hz for the EMG signals. Electrode impedance was kept below 5 kΩ. Sleep staging was performed according to the American Academy of Sleep Medicine criteria [44]. TST and the duration of each sleep stage were derived. In view of the issues surrounding the use of a fixed amplitude threshold to define N3 in older adults which may lead to an underestimation of N3 duration and a consequent overestimation of N2 duration in older adults [45], we analyzed spectral power in the delta range (0.5–4 Hz) and sigma range (12–15 Hz) recorded from C3 and F3.

To assess sleep spindles, we performed automatic sleep spindle detection analyses using the Wonambi Python package, v5.24 (https://wonambi-python. github.io) with an algorithm developed by Wamsley et al. [46] In brief, a Morlet wavelet transformation of artifact-free C3-A2 signal was performed and a moving average was calculated on the wavelet scale corresponding to 12–15 Hz using a 100-ms sliding window. Spindles were detected whenever the moving average exceeded a constant threshold (4.5 times the mean signal amplitude of all artifact-free epochs) for 0.3–3.0 s. We selected this algorithm as a previous study reported that it achieved the best performance compared to several other automated spindle detectors, obtaining the most balanced recall and precision performance and the highest *F*1 score [47]. Spindle count, density (per min), duration (s) and power (µV^2^/Hz) were computed for all NREM epochs from C3-A2 electrodes.

### Statistical analyses

All analyses were performed with SPSS 26.0 (IBM, Chicago, USA). To determine if sleep would benefit the execution of prospective memory intentions compared to the wake group, and whether this would be moderated by age group and relatedness of the intention, we performed a repeated-measures analysis of variance (ANOVA) for the percentage of prospective memory intentions executed with sleep (sleep, wake), age group (young, old) and relatedness (related, unrelated) as predictors. In order to examine if interaction effects would be explained by differences in subjective sleepiness, we performed a repeated-measures analysis of variance (ANOVA) for subjective sleepiness with sleep (sleep, wake), age group (young, old) and session (encoding, retrieval) as predictors. Group contrasts were tested with independent samples and comparisons between conditions were testing using paired-sample *t* tests. For recognition test performance, we assessed the statistical significance of older adults’ performance with a one-sample *t*-test against 100%.

In addition, we performed Pearson correlational analyses in each age group separately to investigate the relationship between sleep parameters and sleep benefits on memory. In order to minimize false positives, we performed correlations in older adults only for memory variables in which an experimental benefit of sleep over wake was established in young adults. We used a Fisher-*z*-transformation to test the significance of the difference between correlations.

Lastly, repeated measures ANOVAs were performed for accuracy and reaction time on the ongoing semantic categorization task in order to compare the relative use of resources for monitoring between younger and older adults. Sleep (sleep, wake), age group (young, old) and session (encoding, retrieval) were included as predictors. Group contrasts were tested with independent samples and comparisons between sessions were tested using paired-sample *t* tests.

## Results

### Age differences in polysomnography sleep parameters

During the night of sleep in the laboratory, older adults obtained significantly less TST, N2 and N3 (*p*s < 0.02, Table 2), and had more WASO (*p* = 0.04) compared to young adults. There were no age group differences in the amount of N1 and REM sleep (*p*s > 0.07), or sleep efficiency (*p* = 0.15). Older adults had significantly reduced NREM delta power and sigma power compared to young adults (*p*s < 0.001), and also had lower spindle count (*p* = 0.01) and shorter spindle duration (*p* = 0.04). There were no significant differences in spindle density and power between age groups (*p* > 0.07).

**Table 2.** Differences in macrostructural sleep parameters between younger and older participants

	Young adults		Older adults		
	Mean	SD	Mean	SD	*p*
TST (min)	486.92	33.01	394.23	64.91	<0.001
N1 (min)	35.24	20.84	41.21	38.67	0.50
N1 %	7.32	4.33	11.63	13.18	0.13
N2 (min)	280.00	39.10	245.93	57.85	0.02
N2 %	57.86	7.31	62.51	10.30	0.08
N3 (min)	67.80	26.04	23.33	31.03	<0.001
N3 %	14.07	5.42	5.69	7.39	<0.001
REM (min)	103.88	30.10	83.76	43.06	0.07
REM (%)	21.38	5.67	21.00	9.97	0.87
WASO (min)	30.06	27.86	50.11	38.06	0.04
Sleep efficiency (%)	89.62	6.11	86.31	9.28	0.15

*p* values from the independent t-tests contrasting the two groups are listed. Characteristics of the young adult group were obtained from a previous study [29].

### Prospective memory performance

**Figure 1.**
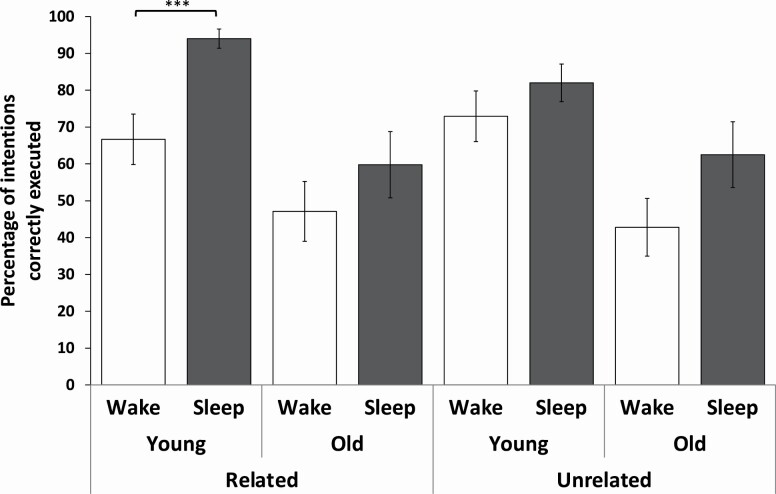
Means and standard errors of the mean for the percentage of related and unrelated intentions that were correctly executed by the young and older adult sleep (grey) and wake (white) groups are plotted. ***p < 0.001.

We found a significant sleep × age group x relatedness interaction (*F* = 6.71, *p* = 0.01, Figure 1). For related intentions, relative to daytime wake, overnight sleep benefitted performance in young adults (*t* = 3.79, *p* < 0.001) but not older adults (*t* = 1.05, *p* = 0.30, age group x sleep interaction: *F* = 1.09, *p* = 0.30). In contrast, unrelated intentions did not benefit from sleep compared to wake in both age groups (young: *t* = 1.07, *p* = 0.29, older: *t* = 1.67, *p* = 0.10, age group × sleep interaction: *F* = 0.53, *p* = 0.47). This indicates that sleep facilitated the spontaneous retrieval of intentions comprising pre-existing associations only in young but not older adults. Lastly, we observed a main effect of age group (*F* = 14.75, *p* < 0.001), which indicated that as expected, young adults performed better on the prospective memory task compared to older adults.

For older adults, 9.14% of prospective memory errors were due to actions performed in response to the wrong cue. The likelihood of this occurring was nearly two times greater for unrelated cue-actions pairs compared to related cue-action pairs (64.7% versus 35.3%). While all young adults scored 100% on the recognition task, older adults did significantly worse (mean ± SEM: 82.35% ± 4.68%, *t* = 3.77, *p* < 0.001), underscoring age-related deterioration in retrospective memory consolidation. Recognition scores for the word pairs were numerically higher in older adults who slept compared to those who remained awake after encoding, but this difference was not significant (mean ± SEM; sleep: 86.98% ± 6.53% versus wake: 78.44% ± 6.68%, *t* = 3.81, *p* = 0.37). Unlike young adults whose prospective memory failures were entirely attributed to forgetting to execute the intention at the right time (i.e. errors in prospective component), older adults were also disadvantaged by their forgetting of the content of the intention (i.e. errors in the retrospective component).

We found no significant interactions or main effects (*p*s > 0.22, Supplementary Table 1) on KSS scores, indicating that prospective memory differences between age groups were not driven by differences in subjective sleepiness.

### Relationship between sleep architecture and prospective memory performance

We previously reported that in young adults, the sleep improvement for the execution of related intentions was associated with the amount of N3 obtained after intention encoding (*r* = 0.46, *p* = 0.02; Table 3). Although there was no sleep versus wake effect on related intentions in older adults, we tested this memory association with N3 duration in older adults to specifically examine if an altered sleep–memory relationship would explain the diminished effect of sleep in older adults. Notably, in contrast with young adults, there was no significant association between N3 and PM consolidation in older adults (*r* = −0.16, *p* = 0.46, *z* = 2.10, *p* = 0.02).

**Table 3.** Pearson correlations between related intentions executed and sleep parameters obtained in the post-encoding sleep period

	Young adults		Older adults	
	*r*	*p*	*r*	*p*
TST (min)	0.18	0.39	0.45	0.03
N1 (min)	0.07	0.75	−0.26	0.23
N1 (%)	0.08	0.72	−0.36	0.09
N2 (min)	−0.35	0.09	0.66	0.001
N2 (%)	−0.40	0.10	0.56	0.01
N3 (min)	0.46	0.02	−0.16	0.46
N3 (%)	0.43	0.03	−0.17	0.44
REM (min)	-0.08	0.68	0.23	0.29
REM (%)	−0.15	0.47	0.10	0.64
C3 NREM delta power (µV^2^/Hz)	0.04	0.87	0.25	0.25
C3 NREM Sigma power (µV ^2^/Hz)	0.04	0.88	0.18	0.40
F3 NREM delta power (µV^2^/Hz)	0.08	0.76	0.20	0.39
F3 NREM Sigma power (µV^2^/Hz)	−0.03	0.91	0.19	0.39
Spindle count (12–15 Hz)	−0.11	0.65	0.22	0.31
Spindle density (per min)	−0.15	0.55	−0.14	0.52
Spindle duration (s)	0.08	0.74	0.04	0.85
Spindle power (µV^2^/Hz)	0.10	0.70	0.07	0.75

Sleep spindles (12–15 Hz) were analyzed with automatic sleep spindle detection using an algorithm developed by Wamsley et al. [46]

We found no significant associations between NREM delta power and the execution of related intentions in either young adults (C3: *r* = 0.04, *p* = 0.87, F3: *r* = 0.08, *p* = 0.76) or older adults (C3: *r* = 0.25, *p* = 0.25, F3: *r* = 0.20, *p* = 0.39). Interestingly, we observed a significant correlation with related intentions and N2 sleep duration in older adults (*r* = 0.66, *p* = 0.001) that was not present in young adults (*r* = −0.35, *p* = 0.09, *z* = −3.70, *p* < 0.001). However, due to our small sample sizes, this finding should be treated with caution. In addition, associations between related intentions and sigma power were not statistically significant in both age groups (*p*s > 0.40). Similarly, associations with related intentions and spindle count, density, duration, and power were not significant in both age groups (*p*s > 0.31). There were no other significant associations with other sleep parameters.

### Semantic categorization task performance

The sleep × age group × session interaction was not significant for accuracy (*F* = 3.73, *p* = 0.06) or reaction time (*F* = 0.31, *p* = 0.58), indicating that for both age groups, sleep did not modulate the additional resources required to monitor for the cue words in the retrieval relative to the encoding sessions. In other words, sleep did not increase the extent of strategic monitoring in either age group.

There were no significant two-way interactions (*p*s > 0.23). However, the main effect of the session was significant for accuracy (*F* = 198.60, *p* < 0.001) and reaction time (*F* = 55.67, *p* < 0.001), indicating that regardless of whether they slept or remained awake, both groups had poorer accuracy and longer reaction times in the retrieval compared to encoding session. This further suggests that both age groups exercised the same level of strategic cue monitoring in the sleep and wake conditions.

## Discussion

While the age-related reduction of sleep’s benefit on declarative memory consolidation has been established with experimental and meta-analytic evidence [7, 9, 48], of recent interest are the factors that may modulate the age effect on sleep-dependent prospective memory. Given the proposition that the relevance of the to-be-remembered material may influence the effect of sleep on memory consolidation [17], the present study investigated prospective memory using a task that mimicked everyday remembering of intentions that involved the actual execution of encoded intentions. While sleep boosted the execution of related intentions in young adults, we did not observe sleep-related improvements for intention execution in our older adult group, suggesting that age impairments seen in sleep-dependent declarative memory consolidation may extend to prospective memory.

Successful execution of an intention involves retrieval of both the prospective component (i.e. self-initiated timely execution) and the retrospective component (i.e. recall of cue-action pairings). Timely execution relies on a self-initiated retrieval triggered by the appearance of the cue, which is then followed by the intentional and directed search for the correct action to perform. Notably, we saw age-related impairments in the sleep-dependent consolidation of both the retrospective (i.e. cue-action pairings) and prospective components (i.e. timely execution) of the PM task. While deficits in the consolidation of the retrospective component are in line with previous studies reporting impairments in the consolidation of word pairs [15, 49, 50] and object locations [51] with increasing age, age differences in the consolidation of the prospective component have been relatively less studied.

Prospective memory retrieval may occur via two pathways: (1) strategic monitoring of the appearance of the cue [29] and/or (2) spontaneous retrieval of the intention. Monitoring behavior may be indexed by reduced accuracy and increased response times on the ongoing semantic categorization in the retrieval session compared to the encoding session. While, as expected, we observed these changes from the encoding to the retrieval sessions, these changes did not differ across sleep and wake retention, suggesting that sleep did not alter monitoring behavior. Also, these changes were similar between the two age groups, indicating that both age groups were monitoring to the same degree. Pertinently, for older adults, changes in semantic categorization performance were similar across both sleep and wake retention intervals, indicating that sleep did not increase their monitoring behavior.

Sleep also did not appear to facilitate spontaneous retrieval in older adults. Spontaneous retrieval, reflected in the successful execution of related (high likelihood of spontaneous retrieval) rather than unrelated intentions (low likelihood of spontaneous retrieval), was not better after sleep versus wake in older adults. In contrast, young adults were more likely to execute related than unrelated intentions after sleep compared to wake, revealing the beneficial effects of sleep in facilitating spontaneous retrieval in this age group.

What mechanisms might underlie this age difference in the effects of sleep on spontaneous retrieval? In young adults, memory reactivation driven by N3 sleep may strengthen the resting activation of related intentions, facilitating spontaneous associative retrieval when the cue is encountered [28, 52]. It has been proposed that an age-related reduction in N3 may drive the impairments in episodic memory consolidation in older adults, and this has been supported by studies reporting that age deficits in sleep-dependent consolidation are proportional to the extent of SWS [53] and frontal SWA age decreases [14, 54]. There is also evidence that the relationship between sleep and memory consolidation may be functionally altered in older adults. For example, the significant and positive associations between N3 amount and episodic memory consolidation seen in young adults may be absent or reversed in older adults [15, 16], suggesting a limited role of N3 sleep for memory that may explain the inconsistent findings in this group. This pattern is consistent with the present findings. In contrast to young adults, we did not find a significant correlation between intention execution and N3 in our older adult sample, suggesting that the function of N3 for prospective memory in this age group may be hampered, rendering it ineffective.

Studies posit that aging may disrupt the precise coupling between field potentials during NREM sleep that drive the hippocampal-neocortical dialogue important for memory consolidation [1]. When slow-wave oscillations no longer effectively entrain sleep spindles, the overnight reactivation and transformation of memory traces may be impeded [50, 55], which may explain why mere availability of SWS might not predict memory performance in older adults.

The reduced ability of atrophied brain areas to support this sleep-driven reactivation process may also contribute to inconsistent findings. For example, even when the reactivation of previously learned material was induced externally, older adults did not show improved post-sleep recall despite oscillatory evidence that cueing was successful [56]. Prospective remembering via spontaneous retrieval has been associated with hippocampal volume [57, 58], and age differences in prospective memory performance have been linked to decreased activation in medial temporal regions [59]. It is possible that although sleep may have boosted representations of the intention, the neural system supporting spontaneous retrieval may not have been sufficiently robust to sustain the requisite activation needed for the intention to be retrieved spontaneously. In other words, age-related brain atrophy may not only impair the generation of quality N3 [14,31], but may also limit the synaptic plasticity needed for prospective memory consolidation.

Interestingly, in older adults, we observed a correlation between related intention execution and N2 sleep. Notably, N2 sleep is relatively preserved with aging and increases approximately 5% between 20 and 70, a reverse pattern from N3, which decreases by 2% per decade [30]. Although this raises the question of whether N2 sleep may be playing a compensatory role for prospective memory, caution must be taken in interpreting this result as we did not find a sleep versus wake benefit for older adults’ prospective memory. Additionally, a recent study has also suggested that REM sleep may play an increased role in prospective memory consolidation with aging [60]. Similar to N2, REM sleep is also relatively maintained with aging and may play a role in supporting cognition as SWS declines [61, 62]. However, we did not find an association with REM sleep in the present work. Future studies in larger samples with age as a continuous variable, and which include a wake control group are needed to address these outstanding issues in sleep research and aging.

## Limitations

Although memory for “intent” versus “content” has been theorized to comprise a performative component that lends it a special salience [63, 64], it is possible that older adults may still have perceived the laboratory-based PM task as relatively abstract. Prior literature has noted an “age-PM paradox,” whereby the age deficit often observed in laboratory-based PM tasks is reversed when PM tasks are instead embedded in natural settings (e.g. remembering to text the experimenter on a specific date) [65, 66]. Notably, a recent study employing a within-subject design using a virtual museum paradigm found sleep improvements in prospective remembering for older adults [34]. Hence, although the present PM task had behavioral relevance, it may still have lacked personal relevance for older participants.

Previous work has suggested that the encoding strength of the initial memory trace may influence sleep consolidation [67, 68]. It is possible that we did not observe significant sleep-dependent memory consolidation effects in our older adult sample because of the encoding quality of intentions. However, weak encoding cannot explain our findings as we ensured accuracy of encoding during the pre-sleep encoding session and also excluded from analyses those who reported confusion regarding task instructions. Further, as participants were only tested once on the pairs after encoding, it is unlikely that associations were robust to the extent that sleep’s effects were rendered redundant [69].

The present study did not include a circadian control group to control for the different times of testing in the sleep and wake groups. Nonetheless, we did not find significant differences in subjective sleepiness measures at encoding or retrieval between wake and sleep groups. Future studies may consider utilizing a nap paradigm where participants may encode and be tested at the same times so as to rule out circadian influences.

## Conclusions

Our findings add experimental evidence to hitherto primarily observational evidence [33] indicating that sleep does not benefit prospective memory consolidation in older adults.

Taken together, the present work contributes to the literature on factors modulating age effects on sleep-dependent memory consolidation. Our findings suggest that aging attenuates sleep-dependent prospective memory consolidation and that this may be linked to changes in the sleep’s role in supporting spontaneous retrieval.

## Supplementary Material

zsab069_suppl_Supplementary_Table_1

## Data Availability

The data underlying this article are available upon reasonable request.
