# Supplementary material for: Sleep-dependent prospective memory consolidation is impaired with aging
Source: Sleep. 2021 Mar 23;44(9):zsab069. doi: 10.1093/sleep/zsab069 (PMC8436136; doi:10.1093/sleep/zsab069)
Supplement: zsab069_suppl_Supplementary_Table_1 [file zsab069_suppl_supplementary_table_1.docx]

**Sleep-dependent prospective memory consolidation is impaired with aging**

Ruth L. F. Leong^1^, June C. Lo^1^, Michael W.L. Chee^1^*

^1^Centre for Sleep and Cognition, Human Potential Program, Yong Loo Lin School of Medicine, National University of Singapore, Singapore

*Corresponding author:

Dr. Michael W.L. Chee

Centre for Sleep and Cognition

NUS Yong Loo Lin School of Medicine,

MD1, 12 Science Drive 2

Singapore 117549

Phone: (+65) 66013199

E-mail: michael.chee@nus.edu.sg

**Supplementary table 1.** Subjective sleepiness in younger and older adult sleep and wake groups at the encoding and retrieval sessions.

|  | **Young adults** | | | | **Older adults** | | | |
| --- | --- | --- | --- | --- | --- | --- | --- | --- |
|  | **Sleep** | | **Wake** | | **Sleep** | | **Wake** | |
|  | **Mean** | **SD** | **Mean** | **SD** | **Mean** | **SD** | **Mean** | **SD** |
| Encoding | 3.24 | 0.88 | 2.96 | 1.08 | 3.82 | 1.11 | 3.54 | 1.02 |
| Retrieval | 2.94 | 1.01 | 2.95 | 0.94 | 3.74 | 1.01 | 3.58 | 0.95 |

Levels of subjective sleepiness measured by the Karolinska Sleepiness Scale.

There were no significant interactions or main effects (ps > 0.22) for KSS scores.
